# Folate deficiency presenting as pyrexia: a case report

**DOI:** 10.1186/1757-1626-1-275

**Published:** 2008-10-26

**Authors:** Aran Singanayagam, Nisal Gange, Anika Singanayagam, Hywel Jones

**Affiliations:** 1 Department of Acute General Medicine, John Radcliffe Hospital, Headley way, Headington, Oxford, OX3 9DU, UK; 2 University of Oxford, Department of Medical Sciences, John Radcliffe Hospital, Headley way, Headington, Oxford, OX3 9DU, UK

## Abstract

Folate deficiency is an uncommon cause of pyrexia. We describe the case of a 29-year-old male who presented with a pyrexial illness subsequently attributed to megaloblastic anaemia secondary to severe folate deficiency, after exclusion of other infective or inflammatory causes. A temperature chart documenting the course of the patient's pyrexia is presented and potential pathophysiological mechanisms are proposed. Folate deficiency is a reversible cause of pyrexia that should be considered in any patient who presents with a pyrexial illness of unknown cause.

## Case presentation

A 29-year-old caucasian male presented to our medical admissions unit complaining of a 6 week history of dyspnoea on exertion, recurrent fevers and sweats. He had a 7 year history of alcohol excess, drinking 3–4 litres of cider per day. He was a smoker of 10 cigarettes per day, but was otherwise fit and well with no significant past medical or family history. The patient reported no other symptoms and did not take any regular medication.

Examination revealed a pulse rate of 124 beats/minute and a blood pressure of 120 mmHg/48 mmHg. He was noted to be febrile with a temperature of 38.8°C. Oxygen saturations were 100% on room air with no signs of respiratory distress. He had visibly pale conjunctiva and a lemon yellow tint to the skin (but no evidence of icteric sclerae). There were no signs of chronic liver disease and no peripheral stigmata of bacterial endocarditis. Cardiovascular examination revealed a collapsing pulse and a soft systolic murmur audible at the left sternal edge. Auscultation of the chest revealed vesicular breath sounds with no added sounds. On abdominal examination there was palpable splenomegaly but not other organomegaly or ascites. Neurological examination was normal with was no evidence of neck stiffness or skin rashes.

Admission blood tests revealed a severe pancytopenia with a raised mean cell volume (see table 1). Also notable was a mild hyperbilirubinaemia but otherwise normal liver function with no evidence of impaired hepatic synthetic function (normal serum albumin and normal prothrombin time).

**Table 1:** Baseline investigations on admission

		Normal range
Haemoglobin (g/dL)	2.8	13.0 – 17.0
Mean cell volume (fL)	112	83 – 105
Platelet count (×10^9^/L)	26	150 – 400
White cell count (×10^9^/L)	2.6	4.0 – 11.0
Neutrophils (×10^9^/L)	1.22	2.0 – 7.0
Lymphocyte (×10^9^/L)	1.24	1.0 – 4.0
Monocytes (×10^9^/L)	0.10	0.2 – 1.0
Reticulocyte count	8.85%	0.5–1.5%
Prothrombin time (secs)	13.9	12.0–14.0
Activated partial thromboplastin time (secs)	35	30.0 – 45.0
Sodium (mmol/L)	130	135 – 145
Potassium (mmol/L)	3.2	3.5 – 5.0
Urea (mmol/L)	6.4	2.5 – 6.7
Creatinine (umol/L)	89	70 – 150
C-Reactive Protein (mg/L)	28	0 – 8
Total bilirubin (umol/L)	28	3 – 17
Alanine transferase (iU/L)	15	10 – 45
Alkaline Phosphatase(iU/L)	110	96 – 280
Gamma GT (iU/L)	24	15 – 40
Albumin (g/L)	38	35 – 50
Thyroid stimulating hormone (mU/L)	3.56	0.35–5.5

Subsequently, serum folate levels were found to be low at 1.2 ug/L (normal range 4–24 ug/L) and vitamin B_12 _levels were low-normal at 202 ng/L (normal range 180–900 ng/L). Iron studies were normal. Initial blood film and subsequent bone marrow examination confirmed a severe megaloblastic picture, consistent with folate deficiency.

Urine dip test and subsequent urine cultures were negative. Three sets of blood cultures were taken from different sites (before commencement of antibiotics) but were all negative after 5 days culture. Chest radiograph was normal with no evidence of focal consolidation. Abdominal ultrasound confirmed splenomegaly but liver size and architecture was normal. A full auto-antibody screen was sent but found to be negative (see table 2).

**Table 2:** Autoimmune screen

		Normal range
Anti-nuclear antibodies	Negative	
Anti smooth muscle antibodies	Negative	
Anti mitochondrial antibodies	Negative	
Anti Gastric parietal cell antibodies	Negative	
Anti LKM antibodies	Negative	
Complement C3	77.5	65–190 mg/dL
Complement C4	20.8	14 – 40 mg/dL
Rheumatoid factor	< 10.0 IU/mL	

## Clinical progression

**Figure 1:**
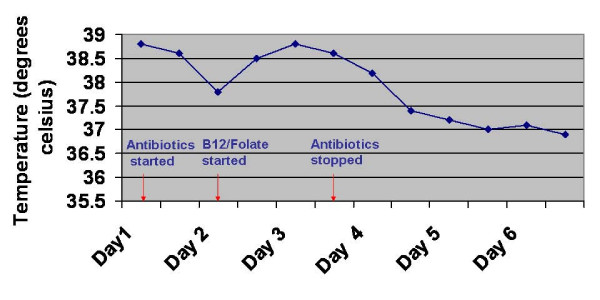
Patient temperature chart.

Initially, the patient was started on intravenous broad spectrum antibiotics as he was presumed to be pyrexial secondary to underlying sepsis of unknown origin. However, despite antibiotics, he did not improve rapidly and continued to remain intermittently pyrexial. Therefore, in the absence of any positive microbiological tests, antibiotics were stopped with continuation of B_12 _and folate supplementation alone. Pyrexia settled on day 4 without any further antimicrobial or anti-inflammatory therapy (see figure 1).

Symptomatically, the patient improved gradually after commencement of B_12 _and folate supplementation on day 2 of admission. Vital signs remained stable throughout the admission. Peripheral blood counts also improved thereafter (see table 3). Subsequent measurement of B_12 _and folate levels at follow-up outpatient appointment showed normalisation.

**Table 3:** Progression of laboratory parameters

	Day 1	Day 2	Day 3	Day 5	Day 7
Haemoglobin (g/dL)	2.8	4.0	5.3	5.8	6.4
Platelet count (×10^9^/L)	26	21	15	20	80
White cell count (×10^9^/L)	2.6	1.62	1.44	3.09	2.77
Neutrophils (×10^9^/L)	1.22	0.87	0.86	1.30	1.05
Sodium (mmol/L)	130	132	132	135	138
Potassium (mmol/L)	3.2	3.0	2.9	3.8	3,8
Creatinine (umol/L)	89	75	64	64	68
C-Reactive Protein (mg/L)	28	29	20	-	22

## Discussion

The case presentation and results of microbiological, immunological and radiological investigations described above support our hypothesis that the occurrence of pyrexia in this patient is attributable directly to the presence of megaloblastic anaemia secondary to folate deficiency. We extensively investigated for other possible infective and inflammatory conditions but found no other contributory cause.

Fever is a feature of megaloblastic anaemia that has been described previously in the literature [1,2] and is thought to be more common in patients with moderate to severe anaemia and thrombocytopenia [3] The level of pyrexia usually correlates with degree of anaemia and resolves one to three days after adequate vitamin supplementation [4], as illustrated by the case we present (see figure 1). Failure of resolution after vitamin supplementation should, however, suggest the probability of an alternative cause for the pyrexia [1].

The exact cause of pyrexia in megaloblastic anaemia is not known and previously it has been hypothesised that it may reflect a defect in oxygenation to the temperature regulatory centres in the brain [5]. However, this theory does not explain why the fever seen in patients with megaloblastic anaemia is not a recognised feature of other forms of anaemia. Another proposed mechanism is that megaloblastic anaemia leads to hyperplasia and thus increased activity within the bone marrow leading to systemic pyrexia [1,5]. The mechanism of how pyrexia is induced by an over-productive marrow is, however, unclear. In previous case series, treatment with B_12 _and folic acid has been shown to cause rapid resolution of fever and this is felt to be due to immediate improvement in ineffective erythropoiesis [1]. This rapid resolution of fever was also seen in the case we describe and further supports our hypothesis of pyrexia caused directly by folate deficiency.

## Conclusion

Megaloblastic anaemia is a rare but reversible cause of pyrexia that should be considered in any patient who presents with a pyrexial illness without other infective or inflammatory source. This is a particularly important consideration as such patients with fever and a pancytopenic peripheral blood picture may initially be wrongly treated with broad spectrum antibiotics as part of a neutropenic sepsis protocol (as demonstrated by the case we describe). Measurement of B_12 _and folate levels should be requested as part of a screen sent for any patient who is pyrexial without an obvious cause.

## Consent

Written informed consent was obtained from the patient for publication of this case report and accompanying images. A copy of the written consent is available for review by the Editor-in-Chief of this journal.

## Competing interests

The authors declare that they have no competing interests.

## Authors' contributions

Ar S performed the literature search and wrote the initial manuscript. NG and An S were major contributors in writing and editing the manuscript. HJ was the supervising consultant involved in the case, made the diagnosis and had the original idea to write up as a case report.
